# Rational Design and Testing of Antibacterial Aloe Vera Hemostatic Hydrogel

**DOI:** 10.3390/gels10060409

**Published:** 2024-06-19

**Authors:** Bryan Shin, Trae Hillyer, Woo Shik Shin

**Affiliations:** 1 Department of Pharmaceutical Sciences, Northeast Ohio Medical University, Rootstown, OH 44272, USA; 2 Solon High School, Solon, OH 44139, USA; 3 University Hospital and Northeast Ohio Medical University Scholarship Program, Rootstown, OH 44272, USA

**Keywords:** aloe vera, hemostatic hydrogel, antibacterial, natural product

## Abstract

Bleeding resulting from surgical procedures or trauma, including gunshot wounds, represents a life-threatening health issue. Therefore, the development of safe, effective, and convenient hemostatic agents is critical in securing the “golden time” to save patients’ lives. Plant-derived compounds and plant extracts have been regarded as promising sources of hemostatic agents in previous studies, regulating hemostatic function with low toxicity and minimal side effects within the human body. Aloe vera-based hydrogels, which are characterized by flexible strength and high functionality, have emerged as a promising platform for wound applications due to their unique biocompatibility features. This study provides a comprehensive exploration of the utilization of thickening agents and natural agents such as xanthan gum, carrageenan, Carbomer, and alginate in applying aloe vera-based hydrogels as a hemostatic. Furthermore, it also tests the use of aloe vera-based hydrogels for therapeutic delivery at wound sites through the incorporation of various antimicrobial agents to extend the utility of the hydrogels beyond hemostasis. Our novel applied research utilizes aloe vera-based hydrogel as an antimicrobial hemostatic agent, providing valuable insights for a wide range of applications and highlighting its potential to enhance hemorrhage control in various emergency scenarios.

## 1. Introduction

Sudden post-traumatic hemorrhages resulting from accidents are the second leading cause of human trauma-related mortality, accounting for 15% of all trauma-related deaths [1,2]. Hemorrhage refers to the escape of blood from blood vessels, and the circulatory system, which comprises vessels throughout the body, circulates blood throughout the body under the pressure of the heart [3]. Typically, the volume of blood in the human body, including both males and females, remains relatively constant at approximately 7–8% of total body weight throughout one’s lifetime [4]. When injury occurs in certain vessels, a space is created for the pressure to escape, leading to the leakage of blood outside the wound [5]. Bleeding, the expulsion of blood from blood vessels, can occur in everyday injuries or medical procedures such as surgery. When blood loss approaches 20% of the total blood volume, the body struggles to maintain normal blood pressure. Under such circumstances, patients typically exhibit unstable physiological symptoms, including dizziness [5,6]. Additionally, excessive bleeding or infection of wounds can potentially be fatal [7,8]. Blood loss exceeding 40% in a short period of time poses a life-threatening risk to the patient. Therefore, regulating the extent of initial hemorrhage is paramount in ensuring the “golden time” for patient treatment [9]. The development of effective and affordable hemostatic agents is imperative for effectively halting bleeding and preventing additional blood loss in individuals who have experienced traumatic injury [10].

Hemostasis refers to the complex process in which platelets and various blood clotting factors present in the blood or plasma cause clot formation through intricate pathways when blood comes into contact with external substances due to vessel injuries, thereby stopping bleeding at the wound site [11,12]. Particularly in cases of excessive bleeding during surgery or emergency situations, the use of effective hemostatic methods is often imperative [12].

There are various hemostatic methods, including mechanical, temperature-based, and chemical approaches [13,14,15,16]. Hemostatic materials are manufactured in various forms tailored for wound treatment purposes, depending on the active components used [17]. Among them, chemical methods, such as the application of hemostatic agents, are representative of treating bleeding areas and halting hemorrhage. Research on hemostatic agents for medical use employing chemical methods has been actively pursued for a considerable period [17]. Hydrogels, by nature, refer to structures possessing a three-dimensional hydrophilic polymer network capable of containing a substantial amount of water or biological fluid [18]. Due to their flexible strength and high functionality, hydrogels closely resemble living tissues, making them widely applicable in the fields of medicine and pharmacy [18,19,20]. In particular, injectable systems utilizing hydrogels enable the design of highly efficient delivery systems to desired locations with simple mixing. Moreover, their fluidity makes them suitable for various applications, including filling deficient areas, and resenting a promising approach across diverse fields.

Plant-derived compounds and extracts are promising sources of hemostatic agents, offering low toxicity and minimal adverse reactions in the human body [21,22,23,24]. Among these, natural ingredients like aloe vera and lotus root stand out for their potential to influence hemostasis [25,26,27]. Aloe vera, a member of the lily family, has a rich history in traditional medicine, and is commonly used as a houseplant and skincare product [25,26,27]. Recognized for its numerous benefits, aloe vera enjoys increasing demand worldwide, particularly in the United States [26]. This growing trend reflects consumer preferences for wellness products with diverse health, pharmaceutical, and cosmetic benefits, supported by aloe vera’s long-established safety profile [26,27,28,29,30]. This suggests future accessibility to hemostatic products based on aloe vera.

Lotus root is rich in tannins and iron, conferring outstanding effects by constricting blood vessels and promoting hemostasis [27]. Tannin, a naturally occurring compound with various reported health benefits, possesses numerous types with antioxidant properties that have been shown to reduce total cholesterol, lower blood pressure, and positively stimulate the immune system [31,32,33,34]. Additionally, the action of tannins associated with vasoconstriction [35,36,37] at the site of injury exhibits excellent hemostatic efficacy, along with rapid wound healing at the injury site. In cases of general trauma, such as oral injuries, the use of tannins can expedite healing and mitigate skin damage [36,37].

In addition to plant-derived substances, there are notable examples of hemostatic efficacy recorded in animal-derived substances as well. One traditional hemostatic method in Korean folk medicine involves the use of cuttlefish bone powder derived from Sepia esculenta. The cuttlefish bone, once dried and ground into a fine powder, is used to manage bleeding from wounds. The application typically involves either sprinkling or topically applying the powder to the affected area. The rationale behind this practice lies in the purported hemostatic effects attributed to the bioactive components inherent in cuttlefish bone. The bones of cuttlefish are composed of an exoskeleton primarily made of chitin [38]. It is known to be harmless to the human body and contains a large amount of calcium carbonate (CaCO_3_). These calcium ions play a crucial role in blood clotting, with carbonate calcium components, in particular, generating heat through a reaction with oxygen in the air, facilitating the evaporation of moisture in the blood, thus imparting hemostatic effects [39]. However, effective scientific approaches are needed for its introduction into modern medicine. We will carefully approach the use of cuttlefish bone powder for hemostasis based on scientific evidence, and ensure both safety and effectiveness.

**Figure 1:**
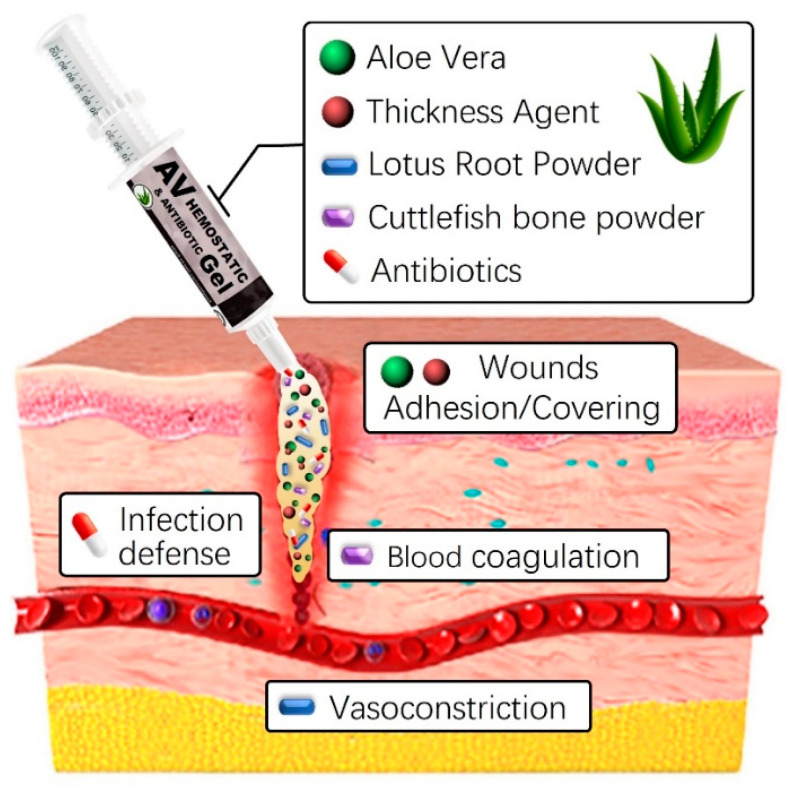
Graphical abstract of aloe vera-based antibacterial hemostatic gel.

The development and use of natural-based hemostatic dressings offer several advantages, including a prompt reduction in bleeding volume and duration at the wound site, as well as lowering infection risks [38,39,40]. Additionally, the straightforward production process and application, along with easy removal post-hemostasis through simple cleansing, provides convenience. In this study, we will combine various natural-based substances, including aloe vera gel and lotus root powder, with thickeners such as carbomer, carrageenan, and alginate to enhance stability. This additional mixture optimizes hemostatic properties while maintaining high viscosity. Although aloe vera gel and lotus root powder already possess skin-friendly qualities, the addition of thickeners and natural preservatives further enhances their interaction with the skin (Figure 1). Furthermore, including a natural preservative like Geogard 221 ensures product stability for at least six months, inhibiting microbial growth [41]. This strengthens the preservation and safety aspects of the product, providing a secure solution for users. Careful research and adjustment of component ratios and interactions are crucial for overall quality improvement and achieving the desired product characteristics. Finding the optimal blend ratio is essential for ensuring high quality and completeness.

By selectively combining plant-derived substances like aloe vera gel and lotus root powder with thickening agents like Carbomer, carrageenan, or alginate, we aim to create an innovative hemostatic agent with enhanced clotting properties. This formulation is expected to offer a safe and effective solution for promoting blood clotting and reducing bleeding in medical applications. To evaluate cost-effectiveness, a detailed cost analysis considering ingredient quantities, manufacturing processes, and potential economies of scale is necessary. However, given the widespread availability and pricing of plant-derived raw materials like aloe vera gel and lotus root powder, we anticipate the developed hemostatic agent to be cost-effective and applicable across various fields. In summary, if proven to be effective and competitively priced, the developed hemostatic agent has the potential to provide significant medical and economic advantages.

## 2. Results and Discussion

### 2.1. Optimization of Skin Adhesive Properties in Aloe Vera-Based Hemostatic Gel

Aloe vera leaves are readily available in our daily lives, and offer the advantage of containing a significantly higher amount of fibrous gel compared to other plants. Filtered through a fine mesh strainer (sieve size 0.4 mm), the remaining impurities were removed, and the aloe vera gel was used in the experiment. Viscosity can also be defined by molecular forces, such as surface fluid friction, which induces resistance to fluid flow. Viscosity is a crucial property used to describe the flow characteristics of hemostatic agents when applied to the site of a skin wound, and a certain level of viscosity is an essential factor in the gel’s ability to adhere to the skin and inhibits the release of blood [42,43].

**Figure 2:**
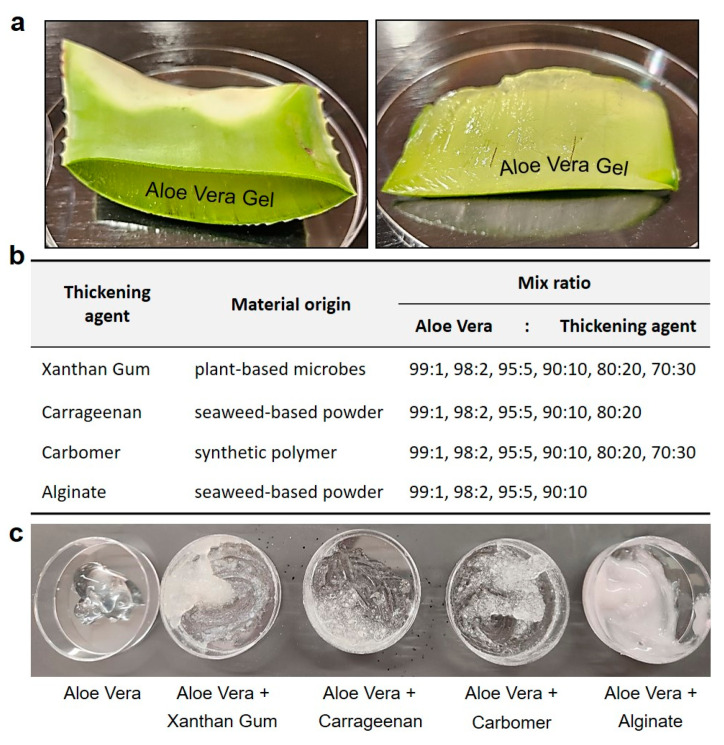
Preparation of aloe vera gel and optimizing the viscosity as hemostatic gel. (**a**) Preparation for the extraction of aloe vera leaves and gel, readily available in our daily lives. (**b**,**c**) In order to optimize the skin viscosity of the hemostatic gel, various edible (xanthan gum, carrageenan) and skin-applicable (Carbomer, alginate) usable thickening agents were added and compared at various ratios.

The first thickening agent consisted of a blend of edible xanthan gum with aloe vera gel extracted from plant leaves. Xanthan gum, derived from plant-based microbes, is a natural compound used in the food and pharmaceutical industries to enhance adhesion and viscosity. The second thickening agent involved the mixture of carrageenan with aloe vera gel. Carrageenan, a rubbery substance obtained from red seaweed, serves as a thickening agent in the production of various foods such as chocolate, ice cream, syrup, and cheese. The third agent mixture was composed of aloe vera gel mixed with Carbomer, a synthetic polymer commonly used as a thickening agent in essences, creams, and lotions, providing a jelly like consistency. The fourth agent comprised aloe vera gel mixed with alginate, a viscous mucilage extracted from seaweeds, widely utilized in cosmetics to ensure viscosity. The initial testing blended hemostatic agents were manufactured by combining each additive with aloe vera gel in ratios of 99:1, 98:2, 95:5, 90:10, 80:20, and 70:30, respectively (Figure 2).

In this manner, initial experiments were conducted using various plant-based components to determine the optimal ratios of each ingredient. Different mixtures were prepared in varying proportions, and tests were conducted to evaluate the skin adhesion effects and texture of each gel. As previously mentioned, thickening agents such as xanthan gum, carrageenan, Carbomer, and alginate were added at predetermined concentrations and ratios, following reported usage amounts and manufacturing guidelines, to progressively enhance viscosity.

To optimize and determine the viscosity for the design of an aloe vera-based hemostatic gel, we systematically screened previously tested aloe vera-based thickening agents and selected them based on their performance rankings. The initial mixture of alginate and aloe vera maintained a viscosity similar to that of lotion but exhibited characteristics more suitable for absorption than skin adhesion. Xanthan gum and carrageenan, commonly used as food thickening agents, initially showed optimal performance in viscosity tests when mixed with aloe vera. However, their slippery texture upon contact with water rendered them unsuitable for skin adhesion, particularly considering that over 80% of blood is composed of water. Finally, Carbomer displayed an appropriate viscosity when initially mixed with aloe vera and showed satisfactory results in skin adhesion tests, even when in contact with flowing water.

Next, we evaluated the practical viscosity of aloe-based mixed gels using Carbomer as the thickening agent. The absolute viscosity was measured using an NDJ-1 Rotor Viscometer, with each sample fluid subjected to the spindle (Rotor 3), rotating at a constant speed of 12 rpm to detect the torque (resistance value) required for rotation [44,45]. To facilitate the comparison of the precise viscosity, and an increase in aloe gel when Carbomer was added, sequential viscosity measurements were conducted on control samples, including water, plant-based oil, detergent, transparent adhesive, and lotion. The absolute viscosity of water was as anticipated, registering at 70 and indicating a very low viscosity. Subsequently, plant-based oil, detergent, glue, and others exhibited absolute viscosity values ranging from 200 to 3300, demonstrating an increase within the expected viscosity range. Additionally, the lotion exhibited a high viscosity and showed similar viscosity to aloe vera. However, the addition of Carbomer as a thickening agent demonstrated a significant increase in the absolute viscosity of aloe vera, aligning with our initial expectations and providing satisfactory results in fulfilling the role of a thickening agent (Table 1).

### 2.2. Hemostatic Ability Pretesting Using the Bleed-Stop Training Kit

**Figure 3:**
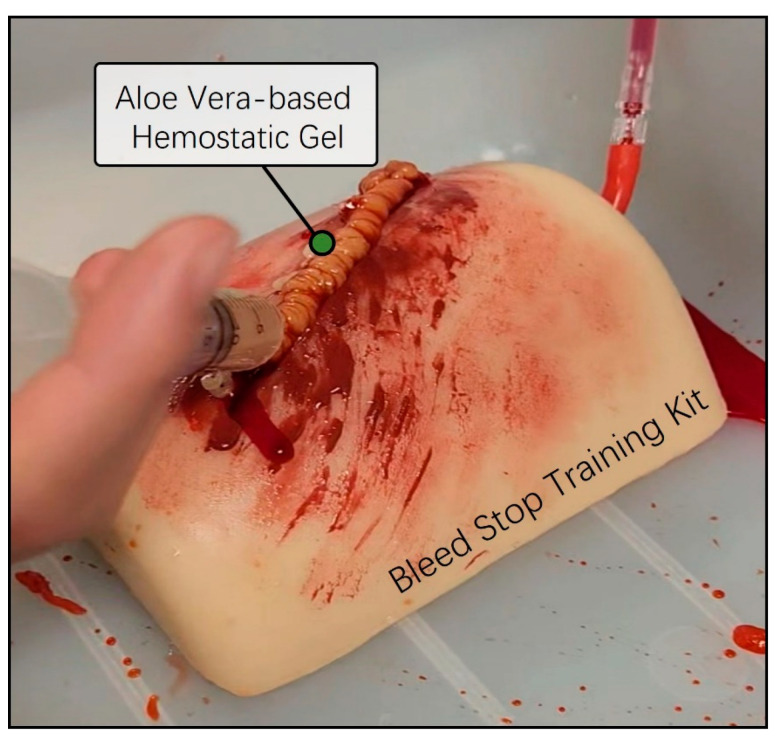
Preassessment of hemostatic ability using a bleed wound control training kit. The Thigh Wound Bleed Control training kit was employed to conduct a preliminary assessment of the hemostatic capabilities of aloe vera-based gel. A simulated scenario was created by allowing model blood contained in a one-liter squeeze bottle to flow under controlled pressure. Using a 25 mL syringe containing aloe vera-based gel, simulations were performed to mimic the continuous flow of blood from a wound, effectively stemming the flow in emergencies.

Next, we conducted a preliminary evaluation of the skin adhesion capability and the anticipated hemostatic efficacy of the previously prepared aloe vera and Carbomer mixed gel using a bleed control training kit. The Bleed Control Kit (MedEduQuest Co., Guangzhou, China) was prepared together with model blood in a 1 L bottle, replicating an emergency bleeding scenario under controlled pressure at a consistent flow rate. The aloe vera-based Carbomer mixed gel was loaded into a 25 mL syringe and was strongly applied to the simulated bleeding wound area. As we expected, the aloe vera and Carbomer mixed hemostatic gel exhibited an appropriate viscosity for the bleed control training kit and effectively arrested the continuous flow of blood without the need for additional physical pressure (Figure 3). Thus, we successfully completed the initial model design of the aloe vera hemostatic gel and validated the desired viscosity for bleeding skin wound areas.

### 2.3. Blood Coagulation Test of Cuttlefish Bone Powder

In essence, by blending powdered cuttlefish bones with aloe vera-based thickening agents in an appropriate ratio, an exceptional trauma dressing gel can be achieved, providing an optimal hemostatic effect. This implies the formation of a hemostatic therapeutic gel, incorporating the powder of cuttlefish bones, known for its blood clotting efficacy, as an external wound treatment in gel form for the human body.

We performed a blood coagulation test on cuttlefish bone powder using a small amount of blood collected from mice (<100 µL). Blood was collected from the venous sinus. The mice used were under anesthesia (2% isoflurane) without any euthanasia or serious trauma, and about 100–150 μL of blood was safely collected. Blood was collected with a syringe (29 G), with gentle pressure on the tail. To achieve hemostasis, blood flow was stopped by applying pressure with sterile gauze. Thirty minutes before blood collection, a local anesthetic (EMLA cream) was also applied to the tip of the tail. The collected blood was immediately refrigerated at 4 °C and stored for the next following experiment.

**Figure 4:**
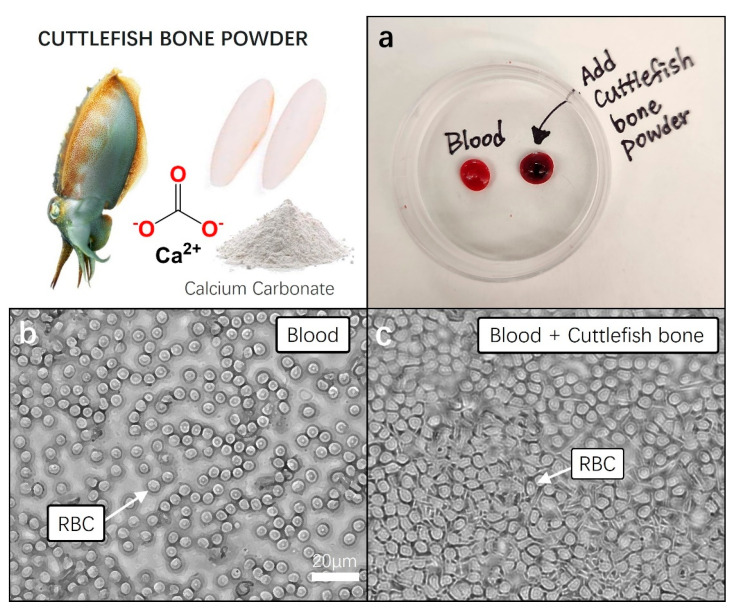
Pretesting blood coagulation properties of cuttlefish bone powder. Prior to incorporating cuttlefish bone powder into aloe vera hemostatic gel, a pre-blood coagulation test was conducted using fresh animal blood. (**a**) Fresh blood and cuttlefish bone powder were added to a 60 × 15 mm Petri dish, gently mixed, and each sample was left undisturbed for 5 min to observe blood coagulation. (**b**,**c**) Each 10 µL blood sample was transferred to a cover slide, and the movement and structure of red blood cells (RBC) were observed under a 20× magnification microscope.

For the blood clotting experiment, we utilized freshly obtained untreated mouse blood as the control group. To assess clotting, 0.05 g of cuttlefish bone powder was added to 50 μL of blood, and gently mixed. In the control group, blood without cuttlefish bone powder, no signs of clotting were observed within the first minute at room temperature. In contrast, the blood treated with cuttlefish bone powder exhibited distinct clotting after approximately 30 s, with nearly complete clot formation in over 90% of the blood samples within 1 min (Figure 4a). We measured the blood clotting time for both cuttlefish bone powder and the control group. The normal blood clotting time results were measured to be approximately 8 to 10 min. The blood clotting index (BCI) for cuttlefish bone powder was calculated as the ratio of the clotting time of the sample (25–35 s) to that of the control group, resulting in a range of 28.5–32. The treatment with cuttlefish bone powder demonstrated a significant blood clotting ability, as we hypothesized.

Following this, each of the blood samples was transferred to cover glass, and clotting states were observed under a microscope with a 40× magnification. In the fresh blood of the control group, we observed the well-maintained, clear, and round shapes of red blood cells. Conversely, in the blood samples showing clotting with the addition of cuttlefish bone powder, a rigid and dense membrane-like structure composed of tightly packed red blood cells was observed. The activation of the fibrin stabilizing factor, a crucial element in blood, was anticipated, indicating the formation of a stable mesh-like structure that confines blood cells, promoting the cross-linking of blood cell molecules and facilitating the formation of stable thrombi (Figure 4b,c).

### 2.4. Vasoconstriction Test of Lotus Root Powder and Its Ingredient (Tannin)

**Figure 5:**
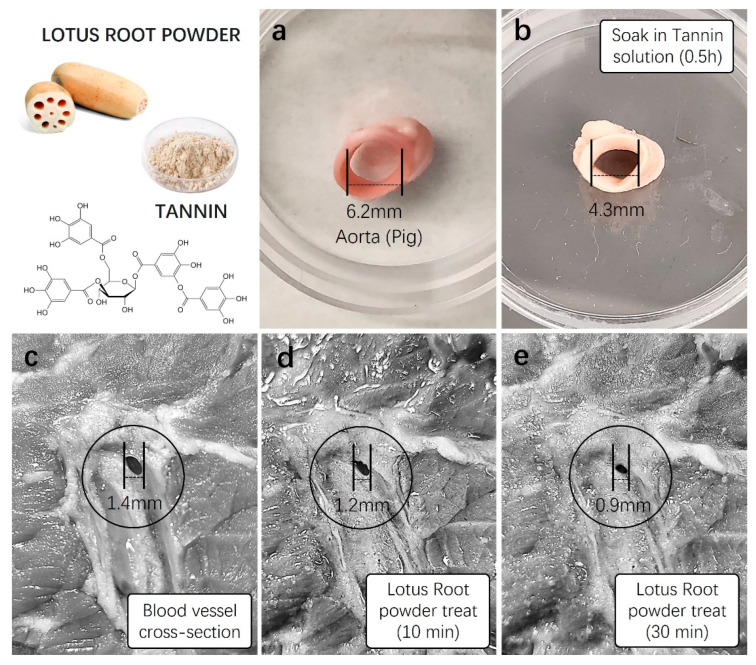
Vasoconstriction testing of lotus root powder. Before incorporating lotus root powder into aloe vera hemostatic gel, preliminary vasoconstriction tests were conducted using a high concentration of tannin identified in lotus root, lotus root powder, and a fresh animal vascular model. (**a**) The diameter of a fresh pig artery was measured, and (**b**) the artery was soaked in a 1 mM tannin stock solution for approximately 30 min. The diameter was then subsequently measured. (**c**) The vascular diameter of fresh pig skin was initially measured (1.4 mm), and (**d**,**e**) solutions of lotus root powder (5 mg/mL) were applied to the vascular area for 10 min (1.2 mm) and 30 min (0.9 mm), respectively, with subsequent diameter measurements.

In order to confirm the hemostatic effect of lotus root, a natural plant-based ingredient rich in tannins and iron, we conducted expected blood coagulation tests using a newly harvested animal blood vessel model after treatment with lotus root powder. We utilized thick thigh skin and tissue samples from pigs, possessing physiological characteristics most similar to humans, and artery samples with appropriate sizes and diameters for vascular contraction experiments. Initially, to confirm the vascular constriction induced by tannins abundant in lotus root, we measured the diameter of fresh pig arteries. Subsequently, the same arterial samples were immersed in a 1 mM tannin solution for approximately 30 min, followed by a re-measurement of the diameter. The results showed a noticeable contraction level, with the artery diameter decreasing from 6.2 mm before tannin treatment to approximately 4.3 mm, as visually confirmed (Figure 5a,b).

Furthermore, the diameter of blood vessels in the subcutaneous tissue of the pig’s thick thigh skin was measured. Subsequently, lotus root powder solution (5 mg/mL) was treated to the vascular region of the tissue, and changes in vascular diameter were measured after 10 and 30 min. The initial measurement of the blood vessel diameter in the subcutaneous tissue of the thigh before treatment with lotus root powder solution was 1.4 ± 0.5 mm (Figure 5c). After 10 min of the treatment, the diameter decreased to 1.2 ± 0.3 mm, a reduction of approximately 0.2 mm. The measurement after 30 min showed a more pronounced contraction, decreasing to 0.9 ± 0.01 mm, indicating a reduction of 0.5 mm (Figure 5d,e). This substantial reduction in vascular diameter was visually observable.

Using blood vessels from the subcutaneous tissue of pig thigh skin, we tested lotus root powder solution and tannin, a major component of lotus root, and showed clear vasoconstriction. These results demonstrated evident vascular constriction, leading us to anticipate significant contributions to hemostatic efficacy upon the addition of these components to aloe vera gel.

### 2.5. Produce of Aloe Vera-Based Hemostatic Gel; Skin Compatibility, Stability, and Shelf-Life Testing

In an attempt to maximize the existing hemostatic effects, including vascular constriction and blood clotting, of the aloe vera-based gel mixed with the previously identified thickening agent, Carbomer, we endeavored to produce an upgraded version of a new compound.

In the first variation, cuttlefish bone powder was added. The calcium carbonate component in squid bone readily absorbs moisture when applied to wounds with bleeding, forming a densely packed membrane structure of red blood cells observed in our experiments. In the second variation, lotus root powder was included. Lotus root powder, rich in tannins and iron, was observed to effectively constrict blood vessels in the wound area, promoting hemostasis. Accordingly, we further conducted the production of a triple composite gel to maximize the vascular constriction effect of lotus root powder and the blood clotting effect of squid bone. The existing aloe vera-based thickening agent was optimized by adding aloe vera gel, thickening agent, lotus root powder, and squid bone in a ratio of 88:2:8:2, achieving the desired gel formulation.

**Figure 6:**
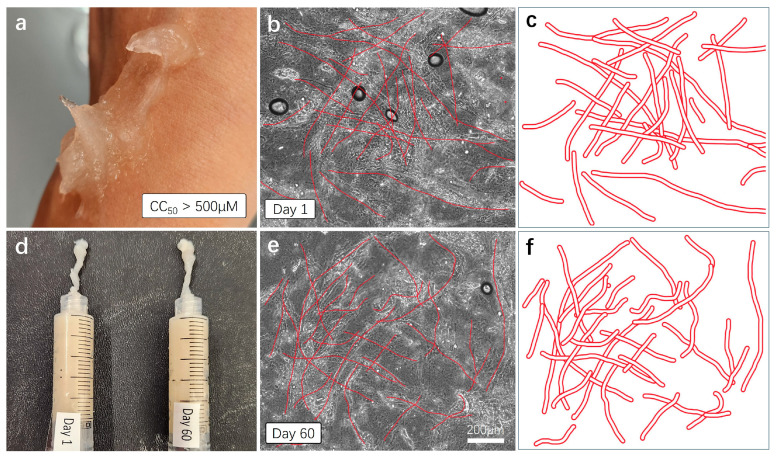
Skin compatibility, stability, and shelf-life testing. (**a**) For the skin compatibility test, the AV-based hemostatic gel was applied to the skin, and the reaction was observed approximately one hour later. (**d**) Mixed hemostatic gels were sealed and stored at room temperature for 60 days. Comparative viscosity and efficacy tests were conducted with freshly made gels. (**b**,**c**,**e**,**f**) Each gel was applied to a cover slice and observed under a microscope with a 20× magnification. The cross-sectional structure of plant-based fibers was schematically represented and re-illustrated with bold red lines.

Furthermore, we verified the stability of the initial product, ensuring thorough mixing of all components. Additionally, we examined whether the gel undergoes degradation or exhibits subtle separation of individual components over time. This is crucial in determining the viability of incorporating our hemostatic gel into future emergency kits (Figure 6).

For the skin compatibility test, we directly applied the aloe vera-based hemostatic gel to the skin and observed skin reactions approximately 1 h later (Figure 6a). As anticipated, no immune reactions, troubles, or damage were observed on the skin, and the gel was easily removed through simple washing with soap. To test the stability and shelf life of the mixed hemostatic gel, we sealed the mixture and stored it at room temperature for 60 days. The gel produced approximately two months prior underwent comparison tests for viscosity, color, and moisture content with a freshly made gel (Figure 6d). The results indicate that the mixed gel stored at room temperature for 60 days show no observable changes in viscosity, color, or moisture content compared to the fresh gel, as observed visually and through touch. For a more detailed comparison, each sample was thinly applied to cover slides and observed under a microscope with a 20× magnification. The dense cross-structure of plant-derived (aloe vera) fibers was easily observed in both samples (Figure 6b,e), and the approximate cross-structure of the fibers was represented again with thick red lines (Figure 6c,f). This cross-fiber-structure based on aloe vera forms a mesh network structure capable of blocking the flow of fluid blood, indicating its essential role in hemostatic action.

### 2.6. Hemostatic Ability Testing Using Thick Animal Skin Subcutaneous Tissue Model

**Figure 7:**
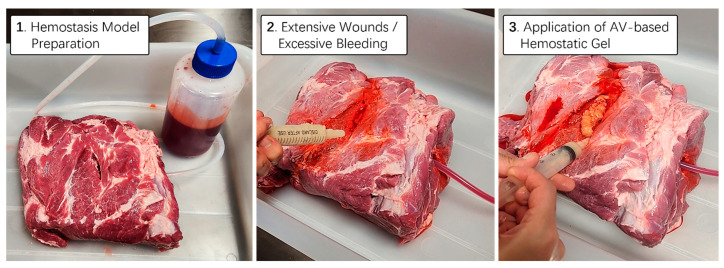
Hemostatic proficiency testing utilizing thick animal skin. A real pig thigh model was employed to evaluate the hemostatic capabilities of aloe vera-based gel. To simulate a bleeding scenario, the blood contained in a 1 L bottle was continuously allowed to flow under controlled pressure. Utilizing a 25 mL syringe containing aloe vera-based hemostatic gel supplemented with cuttlefish bone and lotus root powder, tests were conducted to effectively stem the blood flow from the wound area during emergency situations characterized by persistent bleeding.

To practically assess the hemostatic capability of the aloe vera-based gel, we utilized a real porcine thigh subcutaneous tissue model. Employing the same methodology as in previous artificial bleed wound models, we simulated a bleeding scenario and allowed blood from a one-liter bottle to flow continuously under controlled pressure. Using a 25 mL syringe filled with aloe vera-based hemostatic gel supplemented with squid bone and lotus root powder, we applied it vigorously to the bleeding wound area of the porcine thigh subcutaneous tissue. As anticipated, the mixed hemostatic gel exhibited an appropriate viscosity for the animal’s tissues and skin, effectively blocking the continuous blood flow from the wound without the need for additional physical pressure (Figure 7). Thus, we successfully designed an aloe vera hemostatic gel with upgraded effects on blood coagulation and vascular constriction. Additionally, we verified that the mixed gel could be easily washed off using room temperature water, ensuring ease of removal after application.

### 2.7. Cell-Based Antibiotic Delivery Test from Gel to Skin

The incorporation of antibiotics into hemostatic agents is crucial due to its pivotal role in infection prevention and mitigation of antibiotic resistance. By augmenting hemostatic formulations with antibiotics, one can proactively address the risk of infections at the wound site, contributing to a cleaner healing environment. Additionally, this strategy helps curb the development of bacterial resistance during therapeutic interventions, ensuring the sustained efficacy of antibiotic treatments. This multifaceted approach not only enhances patient safety by reducing the likelihood of postoperative infections, but also holds implications for optimizing wound healing processes in clinical settings.

**Figure 8:**
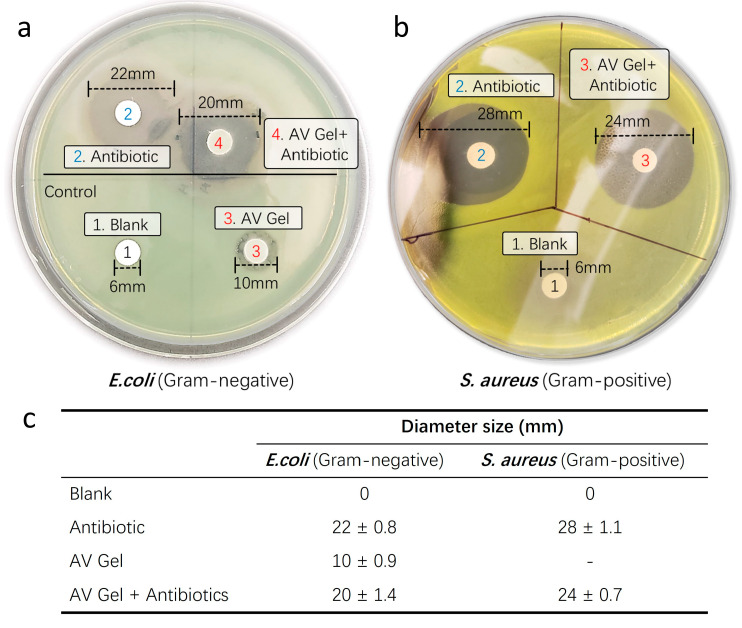
**Antibiotic delivery capability test.** To assess the successful bacterial growth inhibitory capacity of antibiotics incorporated into the hemostatic gel, disk diffusion analyses were conducted using respective Gram-negative (*E. coli*) and Gram-positive (*S. aureus*) strains. (**a**) Gram-negative and (**b**) Gram-positive strains were determined by measuring the diameter of inhibition zones. Untreated disks served as negative controls, while aloe vera gel and liquid antibiotics were utilized as positive controls. (**c**) Summary of the evaluation of the successful antibacterial growth inhibition ability (antibiotic diffusion diameter) of antibiotics contained in the hemostatic gel.

To assess the antibiotic delivery capability and successful inhibition of bacterial growth of our aloe vera-based hemostatic gel, we conducted disk diffusion analyses using respective Gram-negative *Escherichia coli* (*E. coli*) and Gram-positive *Staphylococcus aureus* (*S. aureus*) strains. The disk diffusion assay is a laboratory technique used to evaluate the susceptibility of microorganisms, typically bacteria, to antibiotics or other antimicrobial substances. In this test, paper disks containing a known concentration of an antimicrobial agent are placed on an agar plate inoculated with the microorganism. After incubation, the presence and size of a clear zone of inhibition around the disks indicate the effectiveness of the antimicrobial agent against the tested microorganism. Larger zones generally correspond to greater susceptibility. This assay provides a rapid, qualitative assessment of antimicrobial susceptibility in clinical and research settings (Figure 8a).

After culturing at 37 °C for 24 h, observable colony growth was noted. The inhibitory efficacy of antibiotics incorporated into the hemostatic gel against the Gram-negative bacterium *E. coli* (Figure 8b) and Gram-positive bacterium *S. aureus* (Figure 8c) was determined by measuring the diameter of the inhibition zones. Comparative analysis with liquid forms of Amoxicillin (5 mM) and Meropenem (5 mM) revealed similar inhibition zones for all antibiotic-infused hemostatic gels against bacterial growth when compared to the untreated control discs. Notably, the sole treatment of aloe vera gel exhibited intrinsic antibacterial activity against the Gram-negative bacterium *E. coli* (Figure 8b). Untreated discs served as a control, showing no inhibitory effect against any bacteria. This suggests that aloe vera alone possesses the capability to mitigate the risk of infection at the wound site and, when supplemented with antibiotics in aloe vera gel, may function as a proactive defense against infections.

### 2.8. Animal Skin Wound Model Antibiotic Delivery Test

To explore the therapeutic potential of our aloe vera hemostatic gel augmented with antibiotics as an infection inhibitor, we conducted animal tests using a mouse skin wound infection model. Although the mouse model chosen may not be optimal for testing hemostatic effects due to minimal bleeding at the wound site, our approach allowed for a more rapid assessment of infection inhibition efficacy without significant efforts in addressing potential issues related to solubility, absorption, metabolism, or excretion concerning skin wounds.

**Figure 9:**
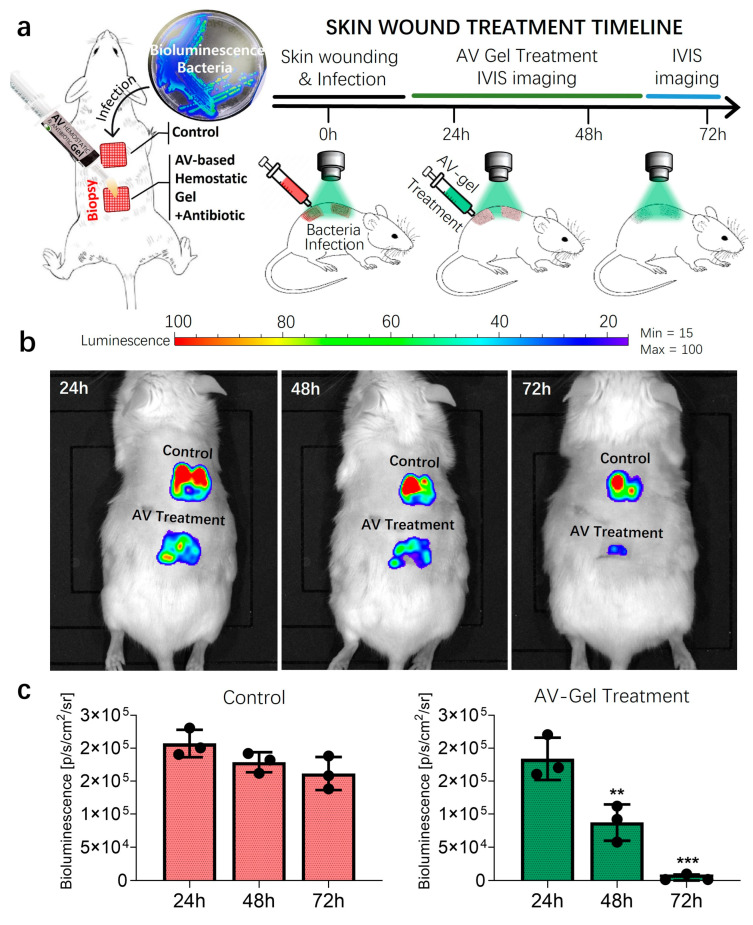
Animal study of mice skin wound models infected with bioluminescent *S. aureus*. (**a**) The treatment scheme of wound infections induced by bioluminescent S. aureus in a mice model following 3 days of treatment for the vehicle control or antibiotic-included AV-gel. A total of 3 Jax Swiss mice, aged 15 weeks, were utilized. (**b**) IVIS imaging of local skin wound infection using bioluminescent *S. aureus* treatment. Three days of infection status comparison was quantified via a bioluminescence signal for each treatment. (**c**) The bar graphs show the quantification of the average bioluminescence intensity emitted by bacteria. Data are presented as mean ± SD (*n* = 3, ** *p* < 0.01, *** *p* < 0.001, one-way ANOVA).

Various strategies exist for managing skin wounds, which can range from superficial abrasions to incisions extending through the subcutaneous muscle and fascia, depending on the depth and regularity of the wound. Opting for a needle scratch method to generate abrasive skin wounds known for inducing stable skin infections, we created two square grids on the mouse dorsum, and inoculated each grid with bioluminescent *S. aureus* (Figure 9a). The use of bioluminescent gene-engineered bacteria enabled real-time tracking of the infection status using the IVIS system [46,47]. IVIS bioluminescent fluorescent images were captured daily before treatment initiation, with DMSO (<5%) as the control group (Figure 9b). Treatment involved daily application of the aloe vera hemostatic gel augmented with antibiotics for three days. Bioluminescent images were acquired daily to track changes in bacterial infection over time.

In both the treatment and control groups, bacterial infections gradually decreased over time, as is evident from the bioluminescent signal intensity. However, wounds treated with the aloe vera hemostatic gel containing antibiotics exhibited a more rapid reduction in bacterial quantity, as represented in Figure 9b,c. The mouse skin wound infection model tests were repeated in triplicate, and the substantial reduction observed in bioluminescent signal variations indicates the effective elimination of the majority of bacteria with the application of the aloe vera hemostatic gel.

## 3. Conclusions

Powerful hemostatic agents effectively control bleeding, reduce the need for blood transfusion, eliminate the necessity for systemic medications to control bleeding, shorten surgical duration, and decrease patient hospitalization costs and duration [48,49]. The development of our novel composite Aloe Vera-based hemostatic agent holds significant potential for use across a broad spectrum of medical applications, including uncontrolled bleeding in various emergency scenarios, encompassing injuries. Its biodegradability, biocompatibility, availability, and low raw material costs enhance its versatility within the medical field. Additionally, the inherent antimicrobial activity and the delivery capability of additional antimicrobial agents offer the potential to prevent secondary infections from contaminated environments, significantly reducing the wound recovery period for patients [50].

The ongoing development of our aloe vera-based antibiotic hemostatic agent represents a practical approach that can provide assistance to numerous soldiers, families, and children currently enduring the hardships of constant conflicts, wars, and upheavals. Formulated by combining various natural ingredients with easily accessible Aloe Vera in everyday life, this antibiotic hemostatic agent offers notable potential for quick dissemination into the emergency kits of those in need, considering its affordable cost, skin safety, user-friendly application and storage, and extended shelf life.

## 4. Materials and Methods

### 4.1. Extraction of Aloe Vera Gel

To obtain a substantial amount of fresh gel, healthy and mature aloe vera (AV) leaves without any damage or discoloration were selected. Before gel extraction, the AV leaves were washed with distilled water and cut horizontally. After removing the yellow latex layer, the collected gel was filtered using a fine mesh strainer (mesh size 0.4 mm) to achieve a more uniform gel consistency. The extracted aloe vera gel was stored in clean, sealed containers in a dark place at 4 °C to maintain its cleanliness and moisture content.

### 4.2. Preparation of Essential Ingredients

Natural premium qualified lotus root powder (harvested and manufactured in Daejeon, Korea) was stored in a 4 °C shady place away from direct sunlight. Cuttlefish bones (Burner World Co., Ltd., Tadley, UK) were precipitated in distilled water for 72 h to remove salt remaining in the cuttlefish bones, and then were soaked in methanol (90%) for 72 h to remove the oily precipitated components. The cuttlefish bones were then washed in distilled water and air-dried at room temperature (20 °C) for 24 h. Completely dried cuttlefish bones were prepared firstly by crushing them using a mortar and pestle and then, secondly, crushing them using a grinder. Powdered cuttlefish bones were stored in a −20 °C freezer to prevent the deterioration of organic matter contained in the bone structure. Xanthan gum (Raw Essentials Co., Bernard, IA, USA), carrageenan (Jacquard Co., Denver, CO, USA), Carbomer (Sanare Co., Denver, CO, USA), and alginate (Smooth-On Inc., Macungie, PA, USA) powder formation were used as thickening agents. Geogard 221 (Kyabo Co., Denver, CO, USA) was used as a natural preservative.

### 4.3. Hemostatic Ability Testing

The Thigh Wound Bleed Control training kit and real pig thigh model were employed to conduct a preliminary assessment of the hemostatic capabilities of aloe vera-based gel. To simulate a bleeding scenario, the blood contained in a 1 L bottle was allowed to flow continuously under controlled pressure. Using a 25 mL syringe containing an aloe vera-based gel, we reproduced hemostatic effects in an emergency situation, characterized by sustained bleeding, and performed tests to effectively block blood flow to the wound site (Supplementary Materials). 

### 4.4. Disk Diffusion Assay

Overnight cultured bacteria of *Escherichia coli* (ATCC 25922) and *Staphylococcus aureus* (Xen 36, ATCC 25923) were diluted in Mueller–Hinton broth until an optical density of 0.08–0.1 at 600 nm was achieved. Kirby–Bauer disks containing either 10 μg amoxicillin/meropenem or 10 μg amoxicillin/meropenem, and including hemostatic gel, were added to the inoculated Mueller–Hinton Agar (MHA) plates and incubated overnight at 37 °C. After 18 h, the diameter of the clear inhibition zone around each disk was measured, along with the plate images.

### 4.5. Luminescence Imaging

Luminescent images were obtained using the IVIS^®^ Lumina XRMS Series III (PerkinElmer, Shelto, CO, USA) in bioluminescence imaging mode. Exposure times were automatically determined using the LivingImage Software version 4.8.2 with medium binning. Mice were kept under anesthesia using the attached XGI-8 Gas Anesthesia System from Caliper LifeSciences, Hopkinton, MA, USA.

### 4.6. In Vivo Test of Animal Skin Wound Model

Bioluminescent *Staphylococcus aureus* Xen 36 was grown overnight in Muller–Hinton broth. The overnight culture was then centrifuged, and the cells were inoculated to the mice in 1× PBS. Three Jax Swiss mice (10 weeks) were anesthetized (3% isoflurane). Mice were then shaved, and the skin was washed with 70% ethanol. A 29 G needle was then used to carefully create the abrasive wound, with caution being paid not to deeply lacerate it. Having created the two sets of wounds (control, treatment), bioluminescent *S. aureus* (30 µL) was added to each wound area. IVIS images were taken after 24 h (start point, t = 0). Treatments were given while animals were anesthetized by adding either the antibiotic-included hemostatic gel or DMSO (2%) directly to the wound area at t = 0, 2, 4, 8, and 24 h. IVIS images were taken once per day prior to any planned treatment. Following the final treatment at t = 24, IVIS images were taken for an additional 2 days.

## Figures and Tables

**Table 1:** Viscosity measurement of aloe-based gel. The viscosity of the aloe-based gel was assessed by comparing the viscosity of gels formulated with aloe gel as the base material and various thickening agents. To facilitate an accurate comparison of viscosity increments, viscosity measurements were conducted on control samples, including water, plant-based oils, detergent, transparent glue, and lotion.

	Water	Oil	Detergent	Glue	Lotion	Aloe Vera	Aloe Vera + Carbomer
Reading (defection angle)	0.7	2 ± 0.1	8 ± 0.1	33 ± 1.7	92 ± 1.4	95 ± 3.1	120 ± 4.3
Absolute viscosity mPa·s @ 22 °C	70	200	800	3300	9200	9500	12000
**Ratio** **(AV: thickening agent)**	**Aloe Vera (absolute viscosity, mPa·s ≈ 9500)**						
	**Xanthan Gum**		**Carrageenan**		**Carbomer**		**Alginate**
	**Absolute Viscosity (Average, mPa·s) @ 22** ** °C**						
99:1	11500		9700		9900		8700
99:2	14000		12500		12000		8100
95:5	35000		31000		27000		5500
90:10	59000		45000		49000		3700
80:20	>60000		>60000		>60000		1400
Adsorption capacity to skin with blood	Not passed		Not passed		Satisfied (Clot formation occurs over 95:5)		Not passed

A rotational viscometer was employed for viscosity calculations, wherein the spindle (rotor 3) was immersed in the sample fluid, rotated at a constant speed (12 rpm), and the torque (defection angle) required for rotation was detected. The absolute viscosity (resistance value) for each fluid was determined by multiplying the reading with the coefficient value of the rotational viscometer.

## Data Availability

All analyzed data are available in the main text, and will also be made available from the corresponding author upon reasonable request.

## Supplementary Materials

The following supporting information can be downloaded at: https://www.mdpi.com/article/10.3390/gels10060409/s1.
